# Transcranial direct current stimulation (tDCS) for improving aphasia after stroke: a systematic review with network meta-analysis of randomized controlled trials

**DOI:** 10.1186/s12984-020-00708-z

**Published:** 2020-07-08

**Authors:** Bernhard Elsner, Joachim Kugler, Jan Mehrholz

**Affiliations:** 1 Department of Public Health, Dresden Medical School, Technical University Dresden, Fetscherstr. 74, 01307 Dresden, Germany; 2 Physiotherapy and Neurorehabilitation, SRH University of Applied Health Sciences Gera, Gera, Germany; 3 Wissenschaftliches Institut, Private Europäische Medizinische Akademie der Klinik Bavaria in Kreischa GmbH, Kreischa, Germany

**Keywords:** Stroke, Aphasia, Transcranial direct current stimulation, Review, Meta-analysis

## Abstract

**Background:**

Transcranial Direct Current Stimulation (tDCS) is an emerging approach for improving aphasia after stroke. However, it remains unclear what type of tDCS stimulation is most effective. Our aim was to give an overview of the evidence network regarding the efficacy and safety of tDCS and to estimate the effectiveness of the different stimulation types.

**Methods:**

This is a systematic review of randomized controlled trials with network meta-analysis (NMA). We searched the following databases until 4 February 2020: Cochrane Central Register of Controlled Trials (CENTRAL), MEDLINE, EMBASE, CINAHL, AMED, Web of Science, and four other databases. We included studies with adult people with stroke. We compared any kind of active tDCS (anodal, cathodal, or dual, that is applying anodal and cathodal tDCS concurrently) regarding improvement of our primary outcome of functional communication, versus control, after stroke. PROSPERO ID: CRD42019135696.

**Results:**

We included 25 studies with 471 participants. Our NMA showed that tDCS did not improve our primary outcome, that of functional communication. There was evidence of an effect of anodal tDCS, particularly over the left inferior frontal gyrus, in improving our secondary outcome, that of performance in naming nouns (SMD = 0.51; 95% CI 0.11 to 0.90). There was no difference in safety between tDCS and its control interventions, measured by the number of dropouts and adverse events.

**Conclusion:**

Comparing different application/protocols of tDCS shows that the anodal application, particularly over the left inferior frontal gyrus, seems to be the most promising tDCS treatment option to improve performance in naming in people with stroke.

## Introduction

Non-invasive brain stimulation (NIBS) is an emerging approach for enhancing neural plasticity and hence rehabilitation outcomes after stroke. There are several stimulation procedures, such as repetitive transcranial magnetic stimulation (rTMS) [1], transcranial direct current stimulation (tDCS) [2–4], transcranial alternating current stimulation (tACS) [5], and transcranial pulsed ultrasound (TPU) [6]. Particularly for rTMS and tDCS a considerable evidence base for NIBS has emerged in the recent years.

tDCS has some advantages when compared to the other stimulation procedures: it is relatively inexpensive, easy to administer and portable, being an ideal add-on therapy during stroke rehabilitation. By applying a weak and constant direct current to the brain tDCS has the ability to either enhance or suppress cortical excitability, with effect lasting up to several hours after the stimulation [7–9]. Hypothetically, this technique makes tDCS a potentially useful tool to for example, post-stroke speech and language therapy (SLT) by modulating inhibitory and excitatory neuronal networks of the affected and the non-affected hemisphere [10, 11]. There are three different stimulation types.

- In anodal stimulation, the anodal electrode (+) usually is placed over the lesioned brain area and the reference electrode over the contralateral orbit [12]. This leads to subthreshold depolarization, hence promoting neural excitation [3].
- In cathodal stimulation, the cathode (−) usually is placed over the non-lesioned brain area and the reference electrode over the contralateral orbit [12], leading to subthreshold polarization and hence inhibiting neural activity [3].
- Dual tDCS means the simultaneous application of anodal and cathodal stimulation [13].

However, there are no clear guidelines regarding to the tDCS type, electrode configuration [14, 15], the amount of current applied and the duration of tDCS, or the question if tDCS should be applied as a standalone therapy or in combination with other treatments [16].

### Rationale

There is so far conflicting evidence from systematic reviews of randomized controlled trials on the effectiveness of different tDCS approaches for improving aphasia after stroke. For example, over the past decade more than 15 randomized clinical trials have investigated the effects of different tDCS stimulation techniques for stroke, and there are at least 23 ongoing trials [17]. However, the resulting network of evidence from randomized controlled trials (RCTs) investigating different types of tDCS (i.e., anodal, cathodal or dual) as well as their comparators like sham tDCS or SLT has not yet been analyzed in a systematic review so far.

A network meta-analysis (NMA), also known as multiple treatment comparison meta-analysis or mixed treatment comparison analysis, allows for a quantitative synthesis of the evidence network. This is made possible by combining direct evidence from head-to-head comparisons of three or more interventions within randomized trials with indirect evidence across randomized trials on the basis of a common comparator [18–21]. Network meta-analysis has many advantages over traditional pairwise meta-analysis, such as visualizing and facilitating the interpretation of the wider picture of the evidence and improving understanding of the relative merits of these different types of neuromodulation when compared to sham tDCS and/or another comparator such as SLT [22, 23]. By borrowing strength from indirect evidence to gain certainty about all treatment comparisons, network meta-analysis allows comparative effects that have not been investigated directly in randomized clinical trials to be estimated and ranked [23, 24].

### Objective

To summarize the evidence network of randomized controlled trials of tDCS for improving functional communication and language function after stroke, as well as its safety.

## Methods

### Protocol and registration

We published a study protocol, which has been registered in the PROSPERO database under the ID CRD42019135696. Our protocol adheres to the PRISMA extension statement for NMA [25].

### Role of the funding source

There was no funding source for this study.

### Eligibility criteria

We included studies with adults who had experienced a stroke. We compared any kind of active tDCS (anodal, cathodal, or dual, that is applying anodal and cathodal tDCS concurrently) for improving our primary outcome of functional communication and our secondary outcome of language function after stroke. Another secondary outcome was safety, measured by the number of dropouts and adverse events. We defined active tDCS as any application of direct current to the skull lasting longer than one minute. This is approximately the time it takes to fade in and fade out the sham application of tDCS in order to produce perceivable sensations on the skin similar to active tDCS [26]. We included all studies with outcome measures evaluating functional communication and for language function. We included all genuine RCTs and genuine randomized controlled cross-over trials which compared tDCS with any other intervention. We analyzed only the first intervention phase of trials with a cross-over design, and assumed between-group differences to be identical to those in trials with a parallel group design. We combined different stimulation durations, different electrical currents applied and different stimulation locations for the same stimulation type (that is anodal, cathodal, or dual tDCS) for our primary analysis. For our a priori defined subgroup analysis we chose the location of stimulation as a potential effect modifier.

### Information sources

We searched the following databases until 4 February 2020: Cochrane Central Register of Controlled Trials (CENTRAL; the Cochrane Library; 2020, Issue 2), MEDLINE (from 1948), EMBASE (from 1980), CINAHL (from 1982), AMED (from 1985), Web of Science (from 1899), Linguistics and Language Behavior Abstracts (LLBA) (from 1973), PsycBITE, speechBITE, Physiotherapy Evidence Database, Rehabdata, COMPENDEX (from 1969) and INSPEC (from 1969). There were no language restrictions. In order to identify further published and unpublished trials, we searched trial registers and reference lists, hand-searched conference proceedings and contacted authors and equipment manufacturers.

### Search

The search strategy for MEDLINE can be found by means of examples in Additional file 1. This search strategy was adapted for the other databases.

### Study selection

One review author (BE) excluded obviously irrelevant studies by reading titles and abstracts. We retrieved the full text of the remaining studies, which were then ranked by two review authors (JK, BE) as relevant, possibly relevant, or irrelevant according to our inclusion criteria. Two review authors (JK, JM) decided whether the possibly relevant publications fitted the PICOS strategy (Patient, Intervention, Comparison, Outcome, Study type) of our research question. We excluded all trials ranked as irrelevant and tested all trials ranked as relevant or possibly relevant for inclusion. Disagreements were resolved by discussions with all review authors.

### Data collection process

Two review authors (BE, JM) independently extracted trial and summary outcome data from the trials.

### Data items

We used checklists to independently assess the following items: (1) methods of random sequence generation, (2) methods of allocation concealment, (3) blinding of outcome assessors, participants and personnel, (4) use of an intention-to-treat analysis, (5) adverse effects and dropouts, (6) important differences in prognostic factors, (7) participants (number, age, time from stroke onset to study entry), (8) comparison (details of interventions in treatment and control groups, duration of treatment and details of co-interventions in the groups) and (9) outcomes at the end of the study.

### Geometry of the network

The geometry of the network characterizes the relation and precision of direct comparisons. At the level of type of intervention, we analyzed what type of tDCS (anodal, cathodal, or dual) was compared with a particular control intervention. The geometry of the network was assessed by generating network graphs [27]. Each type of intervention represents a node in the network. Randomized comparisons between interventions are shown as links between the nodes. The thickness of the line in the network graphs represents the amount of studies included for this comparison. The different colors indicate the median risk of bias among the trials for each of the three dimensions (randomization sequence, concealment of randomization sequence, and blinding) as a covariable at study level in network diagrams.

### Risk of bias within individual studies

We assessed risk of bias of included studies by the Cochrane risk of bias tool at study level [28]. We presented the results for each study in Additional file 5.

### Summary measures

Considering studies that used the same outcome measure for an outcome, we calculated Mean Differences (MD) and their corresponding 95% Confidence Intervals (CI). Including studies that did not use the same outcome measure, but did measure the same underlying construct, we calculated Standardized Mean Differences (SMD) and their corresponding 95% CIs. We expected the number of dropouts and adverse events to be rare and therefore calculated the Risk Difference (RD) and corresponding 95% CIs as the summary measure. For all comparisons we generated contrast-based forest plots. We compiled a relative ranking of the competing interventions on the basis of their surface under the cumulative ranking line (SUCRA) [27]. The SUCRA values give the percentage efficacy of each individual intervention in comparison with an ‘ideal’ treatment. All statistical analyses were performed using the software STATA SE Version 15.0 [29, 30].

### Planned method of analysis

This network meta-analysis was conducted according to a frequentist approach with weighted least squares based on a multivariate regression with random effects. This approach enables adequate consideration of multiple-arm studies and includes restricted maximum-likelihood estimation [31]. The analysis is based on two assumptions: (a) independence of studies and (b) consistency of the underlying effects (transitivity assumption) [32]. We considered the treatment effects to differ between the proof-of-concept trials, and therefore applied a random-effects model to estimate summary treatment effects, based on treatment contrasts. In case there was no source of heterogeneity, for example due to a lack of closed loops, we choose a fixed effects model instead.

### Assessment of inconsistency

To test for possible infringement of the transitivity assumption, we assessed global inconsistency by accommodating a consistency and an inconsistency model [31, 33]. Transitivity means there are no systematic differences among the various arms of the individual studies. At local level we used the node-splitting approach [31, 34]. Alongside the quantitative tests, we performed qualitative verification of the description of the trials included with regard to important effect modifiers.

### Risk of bias across studies

We presented the risk of bias across studies for each comparison separately in our network graphs.

### Additional analyses

We a priori considered the location of stimulation to be a potentially important effect modifier and incorporated it in our prespecified sensitivity analysis.

## Results

### Study selection

We screened 4786 unique records and assessed 80 full-text articles for eligibility. We included 25 trials with 493 participants in a qualitative analysis and 15 studies with 338 participants in a quantitative synthesis (meta-analysis). Additional file 2 shows the flow of studies.

### Study characteristics

Eight of the 25 studies (32%) were RCTs, and the remaining 17 studies (68%) were randomized crossover trials. The sample sizes of the included studies ranged from three [35] to 74 [36]. The mean (SD) sample size was 19 (17) with a median sample size of 13. The mean age of participants ranged from 52 to 68 years. The mean time since stroke ranged from 26 days to 7 years. The current applied ranged from 1 mA to 2 mA, while the number of stimulation sessions ranged from one to 15.

A comprehensive summary of the characteristics of the included trials examining tDCS for improving functional communication and language function, and its safety, can be found in Additional file 3.

### Presentation of network structure

Network graphs presenting the network structure can be found in Additional file 4.

### Summary of network geometry

A total of 112 patients received active tDCS to improve their functional communication (number of studies = 3, number of study arms = 6) [37–39]. The intervention types studied were mostly anodal tDCS (two study arms with 39 participants) [37, 38] or dual tDCS (one study arm with 18 participants) [39]. A total of 55 participants received sham tDCS as a comparator intervention (number of studies = 3) [37–39].

A total of 298 patients received active tDCS to improve performance in naming nouns (number of studies = 11, number of study arms = 25) [11, 36, 37, 39–46]. The intervention types studied were mostly anodal tDCS (eight study arms with 108 participants) [11, 36, 37, 40, 41, 44–46], cathodal tDCS (four study arms with 18 participants) [11, 41, 42, 46], or dual tDCS (two study arms with 27 participants) [39, 43]. A total of 145 participants received sham tDCS as a comparator intervention (number of study arms = 11) [11, 36, 37, 39–46].

A total of 21 patients received active tDCS to improve performance in naming verbs (number of studies = 3, number of study arms = 6) [40, 43, 47]. The intervention types studied were anodal tDCS (one study arm with 4 participants) [40], cathodal tDCS (one study arm with 3 participants) [47], or dual tDCS (one study arm with 4 participants) [43]. A total of 10 participants received sham tDCS as a comparator intervention (number of study arms = 3) [40, 43, 47]. Two study arms with 30 participants used physical rehabilitation comparators like virtual reality and physical therapy [48, 49].

We identified 15 trials (number of study arms = 33) with 338 participants which investigated tDCS for improving functional communication or language function and provided data regarding the safety of tDCS (number of dropouts and adverse effects) [11, 35–37, 39–45, 47, 50]. The intervention types studied were mostly anodal tDCS (10 study arms with 115 participants) [11, 35–37, 39–41, 44, 45], cathodal tDCS (5 study arms with 25 participants) [11, 41, 42, 46, 47], and dual tDCS (three study arms with 37 participants) [39, 43, 51].

Additional file 5 shows a possible approach to presenting data from studies examining the effects of tDCS on functional communication and naming performance, as well as the safety of the interventions (anodal, cathodal, dual or sham tDCS).

### Synthesis of results

**Fig. 1:**
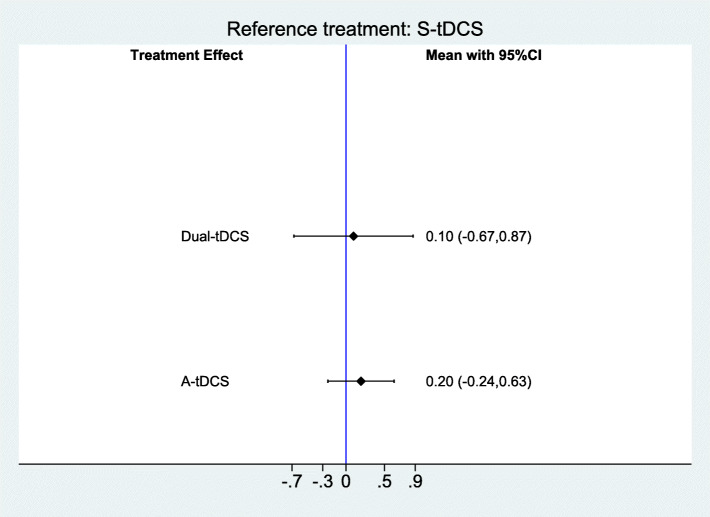
Forest plot of tDCS for improving functional communication in people with aphasia after stroke (3 studies with 112 participants). CI = confidence interval. Sham is the reference category

Figure 1 provides a comparison of effect estimates of different tDCS interventions for improving functional communication (SMD (Standardized Mean Difference) = 0.10; 95% CI − 0.67 to 0.87 for Dual-tDCS and SMD = 0.20; 95% -0.24 to 0.63 for anodal tDCS).

**Fig. 2:**
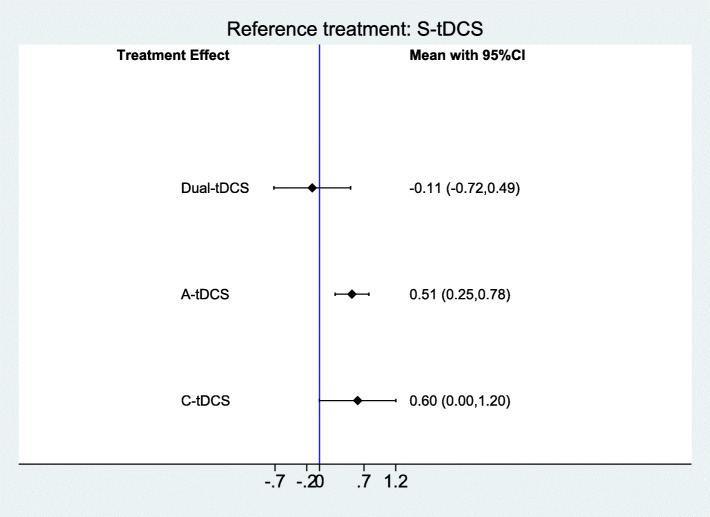
Forest plot of tDCS for improving language function (performance in naming nouns) in people with aphasia after stroke (11 studies with 298 participants). CI = confidence interval. Sham is the reference category

Effect estimates of different tDCS interventions for improving performance in naming nouns can be found in Fig. 2. There was evidence of an effect of anodal tDCS (SMD = 0.51; 95% CI 0.11 to 0.90), but not for Dual-tDCS or cathodal tDCS (SMD = − 0.11; 95% CI − 0.72 to 0.49 and SMD = 0.60; 95% CI − 0.00 to 1.20, respectively).

**Fig. 3:**
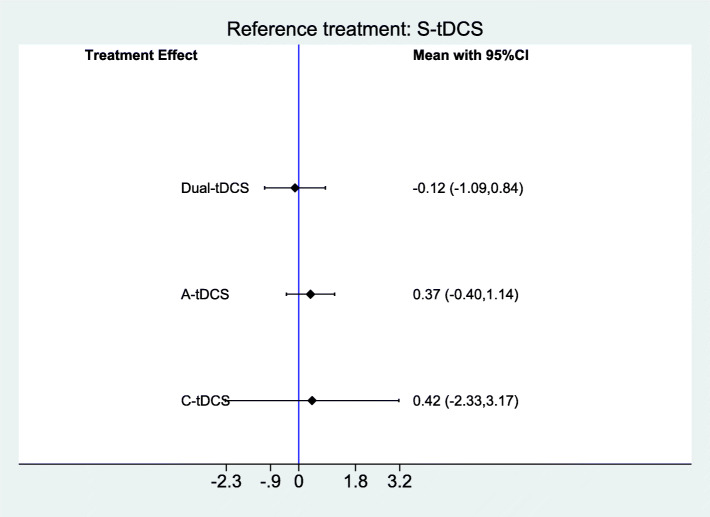
Forest plot of tDCS for improving language function (performance in naming verbs) in people with aphasia after stroke (3 studies with 21 participants). CI = confidence interval. Sham is the reference category

Figure 3 provides a comparison of effect estimates of different tDCS interventions for improving performance in naming verbs. There was no evidence of an effect (SMD = − 0.12; 95% CI − 1.09 to 0.84 for Dual-tDCS, SMD = 0.37; 95% CI − 0.40 to1.14 for anodal tDCS and SMD = 0.42; 95% CI − 2.33 to 3.17 for cathodal tDCS).

**Fig. 4:**
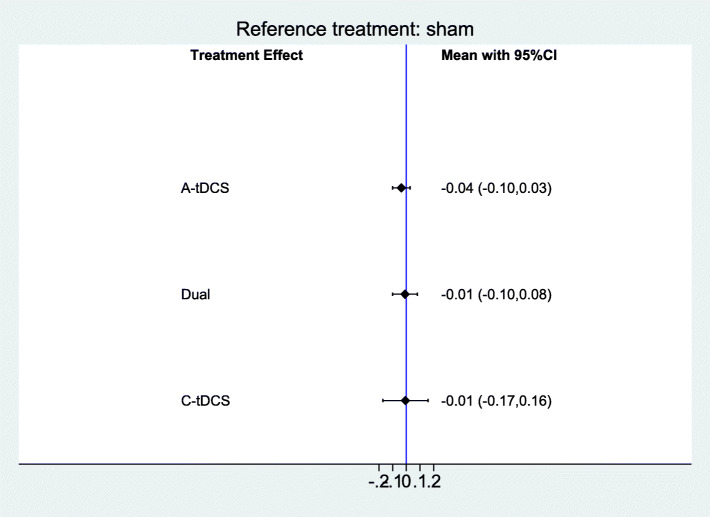
Forest plot of the safety of tDCS for improving functional communication or language function after stroke (15 studies with 338 participants). CI = confidence interval. Sham is the reference category; measure of effect is risk difference (RD)

Figure 4 provides a comparison of effect estimates of different tDCS interventions regarding safety. There was no evidence of a difference in safety between interventions (Risk Difference (RD) = − 0.04; 95% CI − 0.10 to 0.03 for anodal tDCS, RD = − 0.01; 95% CI − 0.10 to 0.08 for Dual-tDCS and RD = − 0.01; 95% CI − 0.17 to 0.16 for cathodal tDCS.

#### SUCRA

Table 1 shows the ranking of the treatments by SUCRA.

**Table 1:** treatment rankings by SUCRA of tDCS for improving functional communication

Outcome	SUCRA
*Functional communication*	
Anodal	0.70
Dual	0.53
Sham	0.28
*Performance in naming nouns*	
Cathodal	0.85
Anodal	0.79
Sham	0.23
Dual	0.14
*Performance in naming verbs*	
Cathodal	0.64
Anodal	0.63
Sham	0.38
Dual	0.35
*Safety*	
Sham	0.66
Cathodal	0.55
Dual	0.52
Anodal	0.28

Treatments are listed in order of relative ranking. The SUCRA, ranging from 0 to 100, describe the percentage efficacy of each individual intervention in comparison with an ‘ideal’ treatment.

### Exploration for inconsistency

Significant inconsistency, which means disagreement between direct and indirect comparisons, was not observed. For the outcomes functional communication and performance in naming verbs there was no source of inconsistency. Formal testing did not detect statistically significant design inconsistency for performance in naming nouns (χ^2^ = 0.51; df = 2; *p* = 0.77) and for safety (χ^2^ = 0.00; df = 2; *p* = 1) and also no local inconsistency.

### Risk of bias across studies

We assessed the risk of bias qualitatively for each outcome by visual inspection of its risk-of-bias graph. We assessed the risk of bias regarding tDCS for improving ADL capacity and arm function and its safety as low to unclear. The risk-of-bias ratings can be found in additional file 5.

### Results of additional analyses

Our prespecified sensitivity analysis on the effects of location and type of stimulation included all 15 studies with 338 participants, which provided the corresponding data. There was evidence that anodal tDCS over the left inferior frontal gyrus (IFG) was the most effective stimulation type for improving performance in naming nouns (Standardized Mean Difference, SMD = 0.51; 95% CI: 0.11 to 0.90). The full results of our subgroup analysis can be found in Additional file 6.

Our meta-regression could not identify any statistically significant effect moderators of tDCS for improving functional communication or language function due to the low numbers of observations.

## Discussion

### Summary of main findings

This systematic review with a network meta-analysis included 25 randomized controlled trials with 471 patients after stroke. Regarding the effect of tDCS on our primary outcome, that of functional communication, an analysis of 3 studies with 112 participants yielded no evidence of effect. Eleven studies with 298 participants examined our secondary outcome, that of language function measured by the performance in naming nouns. We found evidence of an effect of anodal tDCS, whereas for the other interventions dual tDCS, cathodal tDCS, sham tDCS there was no evidence of an effect. Three studies with 21 participants examined the effect of tDCS on language function measured by the performance in naming verbs. We found no evidence of an effect. Finally, our analysis of 15 trials with 338 participants found no statistically significant differences between sham tDCS, anodal tDCS, cathodal tDCS and dual tDCS, regarding our other secondary outcome, that of the safety of tDCS or its control interventions as revealed by the number of dropouts and adverse events. Our prespecified subgroup analysis revealed evidence of an effect in favor of anodal tDCS over the left IFG for improving the performance in naming nouns.

Our network meta-analysis provides new and valuable insights into the relative effects of different types of tDCS. We propose that these analysis can be regarded as a supplement to the previous systematic reviews on the topic [17, 52, 53].

The results of this network meta-analysis in terms of our primary outcome, functional communication, are in line with a recent Cochrane review examining the effects of tDCS in improving aphasia after stroke [17]. Due to the methodological limitations inherent in traditional pairwise meta-analyses the authors of that review could only draw pairwise comparisons, not taking into account the existing evidence network. Furthermore, in order to avoid multiple testing, the authors had to combine treatment groups with different types of tDCS into a single tDCS group, thus maybe masking possible differences between different tDCS types. In a pre-specified formal subgroup analysis for their primary outcome of functional communication, the authors tried to estimate the treatment effects of the different tDCS types (anodal and cathodal). The analysis did not show evidence of an effect for anodal and dual tDCS, which is in accordance with our findings.

Regarding our secondary outcome (i.e., language function), our results are in line with the following systematic reviews with pairwise meta-analysis: Shah-Basak and colleagues (2016) included randomized and observational studies with rTMS and tDCS for improving language function after stroke [52]. They included eight studies examining the effects of tDCS with 140 participants. Their analysis revealed a statistically significant effect of active tDCS at the end of the intervention period (SMD 0.40, 95% CI − 0.28 to 0.51). Another systematic review included 19 studies with an unknown number of participants and qualitative analyses and came to the conclusion that there is emerging evidence in favor of tDCS for improving post-stroke aphasia [53]. The above-mentioned Cochrane review with pairwise meta-analysis also examined the effect of tDCS in naming ability post-stroke [17]. Their analyses revealed a statistically significant effect of active tDCS (with combined treatment groups, anodal, cathodal and dual) at the end of intervention period for improving performance in naming nouns (SMD 0.42, 95% CI 0.19 to 0.66), but not for naming verbs (SMD 0.19, 95% CI − 0.68 to 1.06). This is in accordance with our findings.

### Neurophysiological mechanisms

Until now, there is only poor agreement about the neurophysiology of aphasia recovery and the role of cortical activity in the brain’s hemispheres [15]: so one could argue, that (1) increasing the activation of the dominant (left) hemisphere improves aphasia symptoms (‘vicariation of function’) [54–56], as well as (2) increasing the activation of the non-dominant (right) hemisphere also does [15]. There are further indications, that (3) there is an ineffective maladaptation and overexcitation of the non-dominant (right) hemisphere, which therefore needs to be inhibited (interhemispheric inhibition model), but one could also (4) regard this overexcitation as epiphenomenal [56]. For example, in a cohort study with 14 participants undergoing constraint induced aphasia therapy (CIAT), the clinical gains correlated with increased activity in both hemispheres, as measured by Magnetoencephalography (MEG) [57]. Moreover, the interhemispheric relationship may change over time course of recovery. A systematic review with network meta-analysis examining the effect of tDCS on motor function after stroke came to the conclusion, that cathodal tDCS over the non-lesioned hemisphere resulted in significant gains in activities of daily living (ADL), which would be in favour of the interhemispheric inhibition model [58]. This may imply, that there are different neurophysiological mechanisms of recovery of language and motor abilities after stroke, since in our review we observed the opposite effect of anodal tDCS improving performance in naming nouns, which would be in favour of the ‘vicariation of function’ model. However recently, doubts have been raised about whether these models may be oversimplified or even incorrect and new models have been proposed, such as the bimodal balance-recovery model, which links interhemispheric balancing to the brain’s remaining structural reserve [56].

Additionally, the optimal stimulation paradigm, in terms of the selection of participants likely to benefit from tDCS, the electrode size and location, the amount of direct current applied and the duration of administration remains to be established [14, 56, 59].

### Methodological issues

Besides the above-mentioned neurophysiological explanation for the finding of superiority of anodal tDCS for improving the performance in naming nouns, there might also be methodological reasons. For example, the majority of participants in randomized studies of tDCS for improving language function, measured by the performance in naming nouns, were treated with anodal tDCS (108 out of 157 participants, 71%). Hence, this intervention might have the greatest statistical power in showing evidence, whereas cathodal tDCS was the least powered intervention. Moreover, in this analysis cathodal tDCS ranked higher than anodal tDCS by means of SUCRA which may be due to its higher mean efficacy, although this effect was not statistically significant.

We found evidence of an effect of tDCS for improving performance in naming nouns, but not for improving language function and performance in naming verbs. Since there may be only a weak association between language function (i.e. performance in naming) after stroke and functional communication scores, one could argue that the improvement in language function may not result in a corresponding improvement of performance in everyday communication situations.

### Limitations

To our knowledge, our review, including 25 randomized trials or randomized cross-over trials with 471 participants, is the most comprehensive review so far of the effects and safety of tDCS for improving aphasia after stroke. However, our study has several limitations. These concern the level of individual studies and outcomes in the review as well as that of the review itself. At the level of individual studies, there is the concern about overestimating treatment effects and safety due to unclear risk of bias, and the fact that the reporting of adverse events was often unsatisfactory. Another aspect is that there was methodological and clinical heterogeneity among the included studies regarding study type (the majority of included studies were phase I studies with rather small sample sizes, hence prone to the risk of baseline imbalance), age of the participants, time since stroke, dosage of stimulation, electrode location, base therapy (i.e., concurrent treatment) and level of initial severity. This may be due to the fact that the optimal stimulation paradigm still has to be established, since theoretical assumptions about the interaction between restoration of language function and tDCS-enhanced brain plasticity are still weak. This includes the optimal electrode placement. In popular electrode settings most of the current is redirected by the skin covering the skull, hence being unable to ‘trigger’ neurons effectively [14]. Although tDCS easily could be coupled with novel technologies like, for example computerized picture naming tasks, its added value to rehabilitation outcomes has been limited so far [15]. The bimodal balance recovery model might represent a further step towards a patient-tailored approach to tDCS.

All clinical trials did employ a simplistic dose strategy of tDCS, assuming increased or decreased excitability of stimulated brain areas under the anodal and cathodal electrode, respectively (a detailed qualitative description of interventions can be found in Additional file 3). However, recent dose-response studies suggest that anodal or cathodal tDCS follows a complex, non-linear intensity-dependent effect on neuronal networks [10, 60]. The genotype of patients may also be a predictor of treatment success: a recent randomized study came to the conclusion that people with aphasia with the typical val/val genotype of the brain-derived neurotrophic factor (BDNF) were more likely to benefit from anodal tDCS when compared to atypical BDNF-genotype (Met allele carriers) [61]. However, this has hardly been taken into account in recent studies so far.

Regarding the review level, there is the concern about violating the transitivity assumption, which means that included studies lack comparability. Violating the assumption of transitivity is more likely in larger treatment networks or in systematically different study conditions, like a wide variation in dates of study performance [25]. Neither of these was the case in our analyses. Although our formal analyses regarding inconsistency in the treatment networks were negative, this does not automatically mean that no inconsistency occurred [22]. There partly was an absence of closed loops in the network geometry (for functional communication and performance in naming verbs), which leads to the argument that our analysis is not, in the strictest sense, a NMA or a multiple treatment comparison (MTC), but rather belongs partly to the NMA genus of Adjusted Indirect Treatment Comparison (ITC) [62]. Another point is that network meta-analyses require reasonably homogeneous studies, which is why we restricted our analysis to the post-intervention effects of tDCS. Since stroke is often a chronic disease, future network meta-analyses could also focus on the sustainability of effects of anodal tDCS, acknowledging that the number of published trials that included long-term outcomes is rather small.

## Conclusions

Our network meta-analysis of randomized controlled trials suggests that anodal tDCS, particularly over the left IFG, is the most promising treatment option when tDCS is used to improve language function (measured by the performance in naming nouns) in people with stroke. There is no evidence of an effect of either anodal or any other tDCS stimulation type in terms of improving functional communication or the performance in naming verbs after stroke. No difference regarding safety (in terms of dropouts and adverse events) was seen between different types of tDCS.

Next to improve the methodological quality of the proof-of-concept trials, future trials in humans need to improve reporting the exact dose of tDCS including the electrode montage (electrode size and position) allowing to replicate findings [9]. In particular, the present meta-analysis shows that tDCS trials should improve the methodological quality of research. In particular there is room for improvement with respect to reporting data in randomized cross-over trials for each intervention period separately, allocation concealment, report of drop outs and accompanying intention-to-treat analyses, as well as report of adverse events and long-term outcomes post intervention.

## Supplementary information

**Additional file 1.** Search strategy for MEDLINE.

**Additional file 2.** Study flow chart.

**Additional file 3.** Characteristics of included studies.

**Additional file 4.** Presentation of network structure

**Additional file 5.** Presentation of outcomes of included studies.

**Additional file 6.** Results of subgroup analyses.

## Data Availability

The datasets supporting the conclusions of this article are included within the article and its additional files.

## Abbreviations

95% CI: 95% confidence interval
IFG: Inferior frontal gyrus
M1: Primary motor cortex
MD: Mean difference
NIBS: Non-invasive brain stimulation
NMA: Network meta-analysis
RD: Risk difference
rTMS: Repetitive transcranial magnetic stimulation
SMD: Standardized mean difference
SUCRA: Surface under the cumulative ranking curve
tACS: Transcranial alternating current stimulation
tDCS: Transcranial direct current stimulation
TPU: Transcranial pulsed ultrasound
