# Supplementary material for: Transcranial direct current stimulation (tDCS) for improving aphasia after stroke: a systematic review with network meta-analysis of randomized controlled trials
Source: J Neuroeng Rehabil. 2020 Jul 8;17:88. doi: 10.1186/s12984-020-00708-z (PMC7346463; doi:10.1186/s12984-020-00708-z)
Supplement: Supplementary file 1 — Additional file 1. Search strategy for MEDLINE. [file 12984_2020_708_MOESM1_ESM.pdf]

Additional file 1: search strategy for searching MEDLINE via Ovid

1. exp aphasia/
2. language disorders/ or speech disorders/ or anomia/
3. speech-language pathology/ or exp "rehabilitation of speech and language disorders"/
4. (aphasi$ or dysphasi$ or anomia or anomic).tw.
5. ((speech or language or linguistic) adj5 (disorder$ or impair$ or problem$ or dysfunction)).tw.
6. ((speech or language or linguistic) adj5 (therap$ or train$ or rehabilitat$ or treat$ or remediat$ or intervention$ or pathol$)).tw.
7. or/1-6
8. Electric Stimulation Therapy/
9. Electric Stimulation/
10. Electrodes/
11. (transcranial adj5 direct current adj5 stimulation).tw.
12. (transcranial adj5 DC adj5 stimulation).tw.
13. (transcranial adj5 electric$ adj5 stimulation).tw.
14. (tDCS or A-tDCS or C-tDCS or S-tDCS or electrode$ or anode or anodes or anodal or cathode or cathodes or cathodal).tw.
15. or/8-14
16. 7 and 15
17. exp animals/ not humans.sh.
18. 16 not 17

(this search strategy has been adapted for the other databases)
