# Supplementary material for: Transcranial direct current stimulation (tDCS) for improving aphasia after stroke: a systematic review with network meta-analysis of randomized controlled trials
Source: J Neuroeng Rehabil. 2020 Jul 8;17:88. doi: 10.1186/s12984-020-00708-z (PMC7346463; doi:10.1186/s12984-020-00708-z)
Supplement: Supplementary file 2 — Additional file 2. Study flow chart. [file 12984_2020_708_MOESM2_ESM.pdf]

Additional file 2: PRISMA 2009 Flow Diagram

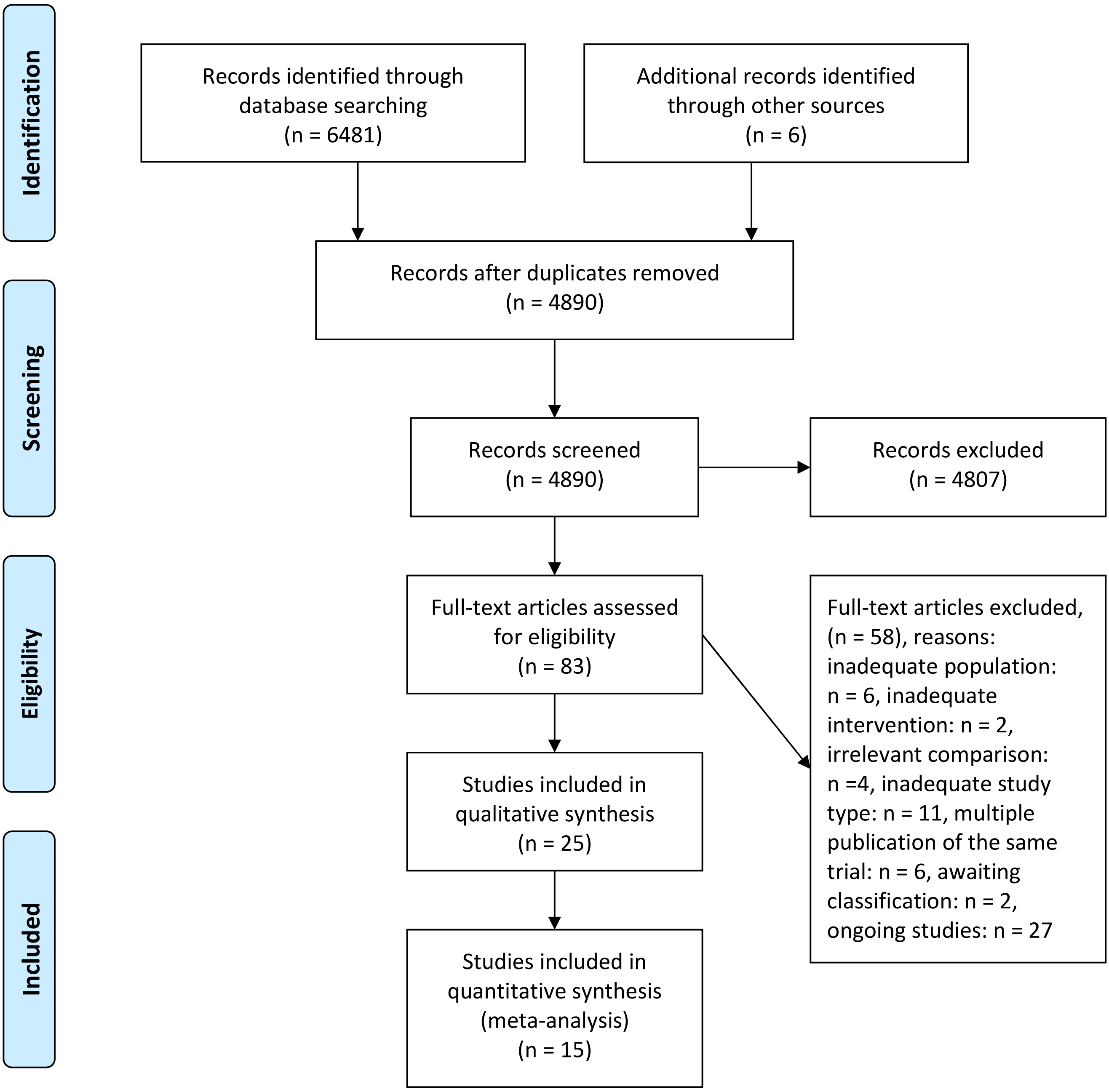

From: Moher D, Liberati A, Tetzlaff J, Altman DG, The PRISMA Group (2009). *P*referred *R*eporting *I*tems for *S*ystematic Reviews and *M*eta-*A*nalyses: The PRISMA Statement. PLoS Med 6(7): e1000097. doi:10.1371/journal.pmed1000097

For more information, visit www.prisma-statement.org.
