# Supplementary material for: Transcranial direct current stimulation (tDCS) for improving aphasia after stroke: a systematic review with network meta-analysis of randomized controlled trials
Source: J Neuroeng Rehabil. 2020 Jul 8;17:88. doi: 10.1186/s12984-020-00708-z (PMC7346463; doi:10.1186/s12984-020-00708-z)
Supplement: Supplementary file 3 — Additional file 3. Characteristics of included studies. [file 12984_2020_708_MOESM3_ESM.pdf]

Additional file 3: characteristics of studies examining the effects of tDCS on functional communication, language function and safety

Study-ID	Design and sample size	EXP mean age (SD), years	CTL mean age (SD), years	EXP mean time post-stroke (SD)	CTL mean time post-stroke (SD)	Stimulation variables	Control Intervention	Base treatment	Outcomes
Baker 2010	RCOT (n=10)	66 (11) years		65 (68) months		20 min with 1 mA of anodal tDCS with the anode placed over the most active area of the left frontal cortex<br>Reference electrode position: on the right shoulder<br>Each treatment was delivered on 5 consecutive working days, separated by 7 days wash-out period	Sham tDCS (1 mA for 30 s)	Computerised anomia training (picture naming)	Naming accuracy
Branscheidt 2018	RCOT (n=16)	61 (10) years		23 (18) months		20 min with 2 mA of anodal tDCS with the anode placed over the left M1<br>Reference electrode position: over the right supraorbital region<br>Each treatment was delivered once, separated by 7 days wash-out period	Sham tDCS (2 mA for 30 s)	Lexical decision tasks with pseudo words and existing words	Naming accuracy, response time
Dos Santos 2017	RCOT (n=13)	Not described		Not described		20 min with 2 mA of anodal tDCS with the anode placed over the left IFG<br>Reference electrode position: over right IFG<br>Number of treatment sessions and wash-out period not described	Sham tDCS (2 mA for 20 s) or TMS (1 Hz for 20 min) or Sham TMS (1 Hz for 20 s)	Boston Naming Test	Naming accuracy, response time, picture-naming strategy, response time strategy, total response time
Fiori 2013	RCOT (n=7)	58 (10) years		33 (28) months		20 min with 1 mA of anodal tDCS with the anode placed either over the left IFG or STG<br>Reference electrode position: over the right supraorbital region<br>Each treatment was delivered on 10 consecutive sessions, separated by 6 days wash-out period	Sham tDCS (1 mA for 30s)	Computerised anomia training (video naming)	Naming accuracy
Flöel 2011	RCOT (n=12)	52 (9) years		84 (65) months		20 min with 1 mA of either anodal or cathodal tDCS over the right temporo-parietal cortex<br>Reference electrode position: over the right supraorbital region	Sham tDCS (1 mA for 30s)	Anomia training (picture naming)	Naming accuracy

						Each treatment was delivered on 10 consecutive sessions, separated by 6 days wash-out period			
Fridriksson 2018	RCT (n=74); Anodal: 34 Sham: 40	60 (11) years	60 (10) years	44 (45) months	40 (35) months	20 min with 1 mA of anodal tDCS over the most active cortex during naming identified by fMRI<br>Reference electrode position: over the right supraorbital region<br>Each treatment was delivered 2 to 5 times per week for 3 weeks, separated by 7 days wash-out period	Sham tDCS (1 mA for 30s)	Computerised anomia training (picture naming)	Naming accuracy
Guillouet 2020	RCOT (n=10)	54 (14) years		18 (21) months		20 min with 2 mA of dual tDCS over the left IFG and the cathode over the right IFG<br>Each treatment was delivered on 5 consecutive sessions, separated by 7 days wash-out period	Sham tDCS (1 mA for 30s)	Tailored speech and language therapy	Functional communica tion, naming performanc e
Kang 2011	RCOT (n=10); Cathodal: 5 Sham: 5	62 (9) years		52 (69) months		20 min with 2 mA of cathodal tDCS over the right IFG<br>Reference electrode position: over the left supraorbital region<br>Each treatment was delivered on 5 consecutive sessions, separated by 7 days wash-out period	Sham tDCS (1 mA for 30s)	Computerised anomia training (picture naming)	Naming accuracy, response time
Marangolo 2011	RCOT (n=3)	66 (3) years		22 (22) months		20 min with 1 mA of anodal tDCS with the anode placed either over the left IFG<br>Reference electrode position: over the right supraorbital region or shoulder<br>Each treatment was delivered on 5 consecutive sessions, separated by 6 days wash-out period	Sham tDCS (1 mA for 30s)	Tailored speech and language therapy	Naming accuracy
Marangolo 2013a	RCOT (n=12)	60 (8) years		37 (22) months		20 min with 1 mA of anodal tDCS with the anode placed either over the left IFG or STG<br>Reference electrode position: over the right supraorbital region<br>Each treatment was delivered on 10 consecutive sessions, separated by 6 days wash-out period	Sham tDCS (1 mA for 30s)	Computerised anomia training (video naming)	Amount of stated content units, verbs and sentences
Marangolo 2013b	RCOT (n=8)	55 (9) years		29 (24) months		20 min with 2 mA of dual tDCS with the anode over the left IFG and the cathode over the right IFG<br>Each treatment was delivered on 10 consecutive sessions, separated by 14 days wash-out period	Sham tDCS (2 mA for 30s)	Audiotape based word repetition training	Naming accuracy, response time
Marangolo 2013c	RCOT (n=7)	62 (10) years		41 (27) months		20 min with 1 mA of anodal tDCS with the anode placed either over the left IFG or STG<br>Reference electrode position: over the right supraorbital region	Sham tDCS (1 mA for 30s)	Computerised anomia training (video naming)	Naming accuracy, response time

						Each treatment was delivered on 10 consecutive sessions, separated by 6 days wash-out period			
Marangolo 2018	RCOT (n=12)	58 (8) years		22 (7) months		20 min with 2 mA of cathodal tDCS with the cathode placed either over the right cerebellum<br>Reference electrode position: over the right shoulder<br>Each treatment was delivered once, separated by a wash-out period of unknown duration	Sham tDCS (2 mA for 30s)	Verb generation and verb naming task	Naming accuracy, response time
Meinzer 2016	RCT (n=26); Anodal: 13<br>Sham: 13	59 (13) years	61 (12) years	54 (22) months	37 (26) months	20 min with 1 mA of anodal tDCS over the left M1<br>Reference electrode position: over the right supraorbital region<br>Treatments were delivered two times per day on 4 days per week for 2 weeks	Sham tDCS (1 mA for 30s)	Computer-assisted naming treatment with the 'vanishing cues' approach	Functional communica tion (CETI), naming accuracy
Monti 2008	RCOT (n=8)	60 (12) years		47 (23) months		10 min with 2 mA of either anodal or cathodal tDCS over the left IFG<br>Reference electrode position: over the right shoulder<br>Each treatment was delivered once with at least 7 days wash-out period	Sham tDCS (2 mA for 10s)	Computerised anomia training (picture naming)	Naming accuracy, response time
Pestalozzi 2018	RCOT (n=14)	57 (9) years		35 (29) months		20 min with 1 mA of anodal tDCS over the left IFG<br>Reference electrode position: over the right supraorbital region<br>Each treatment was delivered once with at least 7 days wash-out period	Sham tDCS (2 mA for 1 min)	Picture naming task	Naming accuracy, response time
Polanowska 2013	RCT (n=37); Anodal: 16<br>Sham: 16	58 (10) years	61 (12) years	56 (45) days	64 (43) days	10 min with 1 mA of anodal tDCS over the left IFG<br>Reference electrode position: over the right supraorbital region<br>Treatments were delivered 5 times per week for 3 weeks	Sham tDCS (1 mA for 25s)	Computerised anomia training (picture naming)	Naming accuracy
Rosso 2014	RCOT (n=25)	57 (18) years		15 (20) months		15 min with 2 mA of cathodal tDCS with the cathode placed either over the right cerebellum<br>Reference electrode position: over the right shoulder<br>Each treatment was delivered once, separated by a wash-out period of 2 hours	Sham tDCS (1 mA for 16s)	Computerised anomia training (picture naming)	Naming accuracy
Shah-Basak 2015	RCOT (n=7)	64 (9) years		31 (30) months		20 min with 2 mA of either cathodal tDCS with the cathode placed either over the right or left frontal cortex<br>Reference electrode position: over the contralateral mastoid<br>One of the four active treatments and one of the two sham treatments was delivered 5 times per week for 2 weeks	Sham tDCS either over the left or right frontal cortex (2 mA for 1 min)	Constraint induced language therapy	WAB, WAB-AQ

Spielmann 2016	RCT (n=58); Anodal: 26 Sham: 32	58 (10) years	60 (10) years	1.4 (0.5) months	1.6 months (0.7)	20 min with 1 mA of anodal tDCS over the left IFG<br>Reference electrode position: over the right supraorbital region<br>Treatments were delivered on 5 consecutive sessions	Sham (1 mA for 30 s)	Word-finding therapy	Functional communication (ANELT), naming accuracy
Spielmann 2018	RCOT (n=13)	53 (11) years		49 (48) months		20 min with 1 mA of anodal tDCS with the anode placed either over the left IFG or STG<br>Reference electrode position: over the right supraorbital region<br>Each treatment was delivered once, separated by >3 days wash-out period	Sham (1 mA for 60 s)	Word-finding therapy	Naming performance
Turkeltaub 2017	RCT (n=38); Dual: 24 Sham: 14	60 (10) years	60 (9) years	Not stated		Unknown duration with unknown current dosage of dual tDCS with the anode placed over the left temple and the cathode over the right	Sham tDCS (dosage not described)	Speech and language treatment	Functional communication (BDAE), naming accuracy
Vila-Nova 2019	RCOT (n=12)	58 (13) years		37 (17) months		20 min with 1 mA of anodal tDCS with the anode placed over the left IFG<br>Reference electrode position: over the right supraorbital region<br>Each treatment was delivered on 5 consecutive days, separated by 2 days wash-out period	Sham (1 mA for 5s)	Picture naming task	Naming performance
Volpe 2014	RCOT (n=15)	Between 18 and 65 years (n=7), above 65 years (n=8)		> 6 months		Unknown duration with unknown current dosage of anodal tDCS, electrode positioning not described	Sham tDCS (dosage not described)	Computerised aphasia therapy once	Naming accuracy
You 2011	RCT (n=21); Anodal: 7 Cathodal: 7 Sham: 7	68 (11) years	63 (10) years	26 (6) days	25 (9) days	30 min with 2 mA of either anodal tDCS over the left STG or cathodal tDCS over the right STG<br>Reference electrode position: over the forehead<br>Each treatment was delivered on 10 consecutive sessions	Sham tDCS (2 mA for 60s)	Conventional speech and language therapy	WAB-AQ

ANELT: Amsterdam-Nijmegen Everyday Language Test, BDAE: Boston Diagnostic Aphasia Examination, CETI: Communicative Effectiveness Index, fMRI: functional Magnetic Resonance Imaging, IFG: inferior frontal gyrus, M1: primary motor cortex, mA: milliampere, RCOT: randomised cross-over trial, RCT: randomised controlled trial, SD: standard deviation, SIS: Stroke Impact Scale, STG: superior temporal gyrus, tDCS: transcranial direct current stimulation, WAB: Western Aphasia Battery, WAB-AQ: Western Aphasia Battery, Aphasia Quotient
