# Supplementary material for: Transcranial direct current stimulation (tDCS) for improving aphasia after stroke: a systematic review with network meta-analysis of randomized controlled trials
Source: J Neuroeng Rehabil. 2020 Jul 8;17:88. doi: 10.1186/s12984-020-00708-z (PMC7346463; doi:10.1186/s12984-020-00708-z)
Supplement: Supplementary file 4 — Additional file 4. Presentation of network structure [file 12984_2020_708_MOESM4_ESM.pdf]

Additional file 4: presentation of network structure

Presentation of network structure

Figure 1 shows a network graph comparing anodal, cathodal, and dual tDCS with their control interventions for improving functional communication after stroke. Figure 2 shows a network graph comparing anodal, cathodal, and dual tDCS with their control interventions for improving language function (naming nouns) after stroke and Figure 3 for improving naming verbs. Figure 4 shows a network graph comparing tDCS with their control interventions regarding safety (measured by the number of dropouts and adverse events).

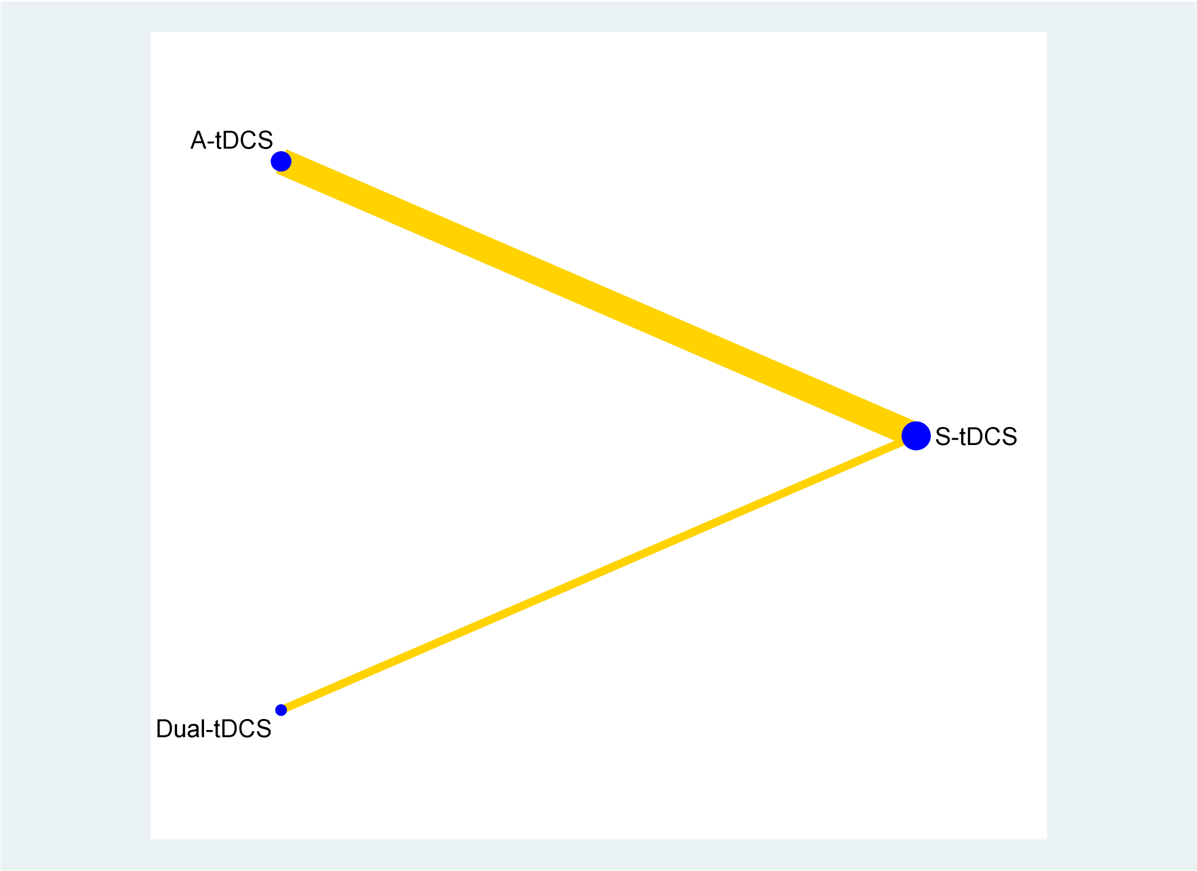

Figure 1: Network graph of tDCS for improving functional communication in people with aphasia after stroke. The thicker the blue bubble, the higher the sample size of the corresponding intervention and the thicker the edge, the lower the standard error of this comparison. Colors of edges indicate risk of bias for this comparison (green = low, yellow = unclear and red = high risk of bias).

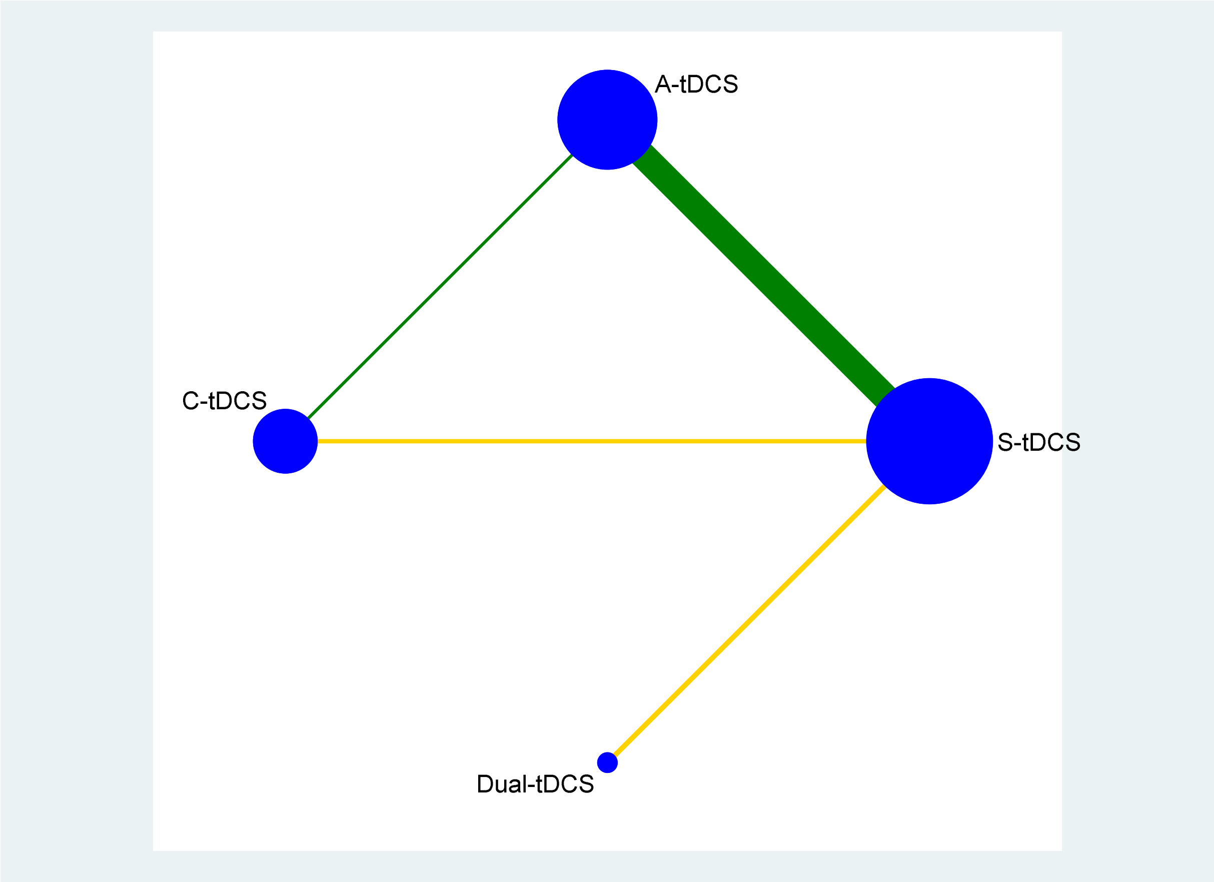

Figure 2: Network graph of tDCS for improving language function (measured by the performance in naming nouns) in people with aphasia after stroke. The thicker the blue bubble, the higher the sample size of the corresponding intervention and the thicker the edge, the lower the standard error of this comparison. Colors of edges indicate risk of bias for this comparison (green = low, yellow = unclear and red = high risk of bias).

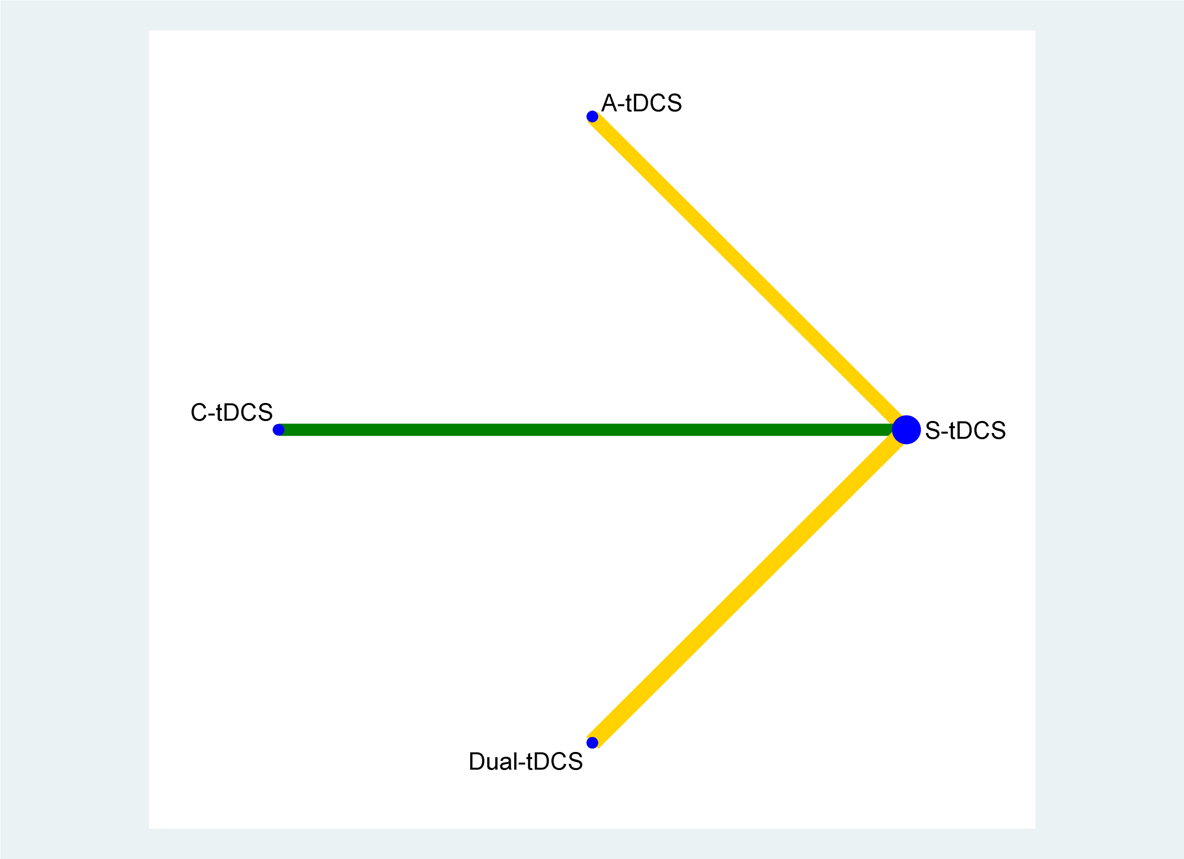

Figure 3: Network graph of tDCS for improving language function (measured by the performance in naming verbs) in people with aphasia after stroke. The thicker the blue bubble, the higher the sample size of the corresponding intervention and the thicker the edge, the lower the standard error of this comparison. Colors of edges indicate risk of bias for this comparison (green = low, yellow = unclear and red = high risk of bias).

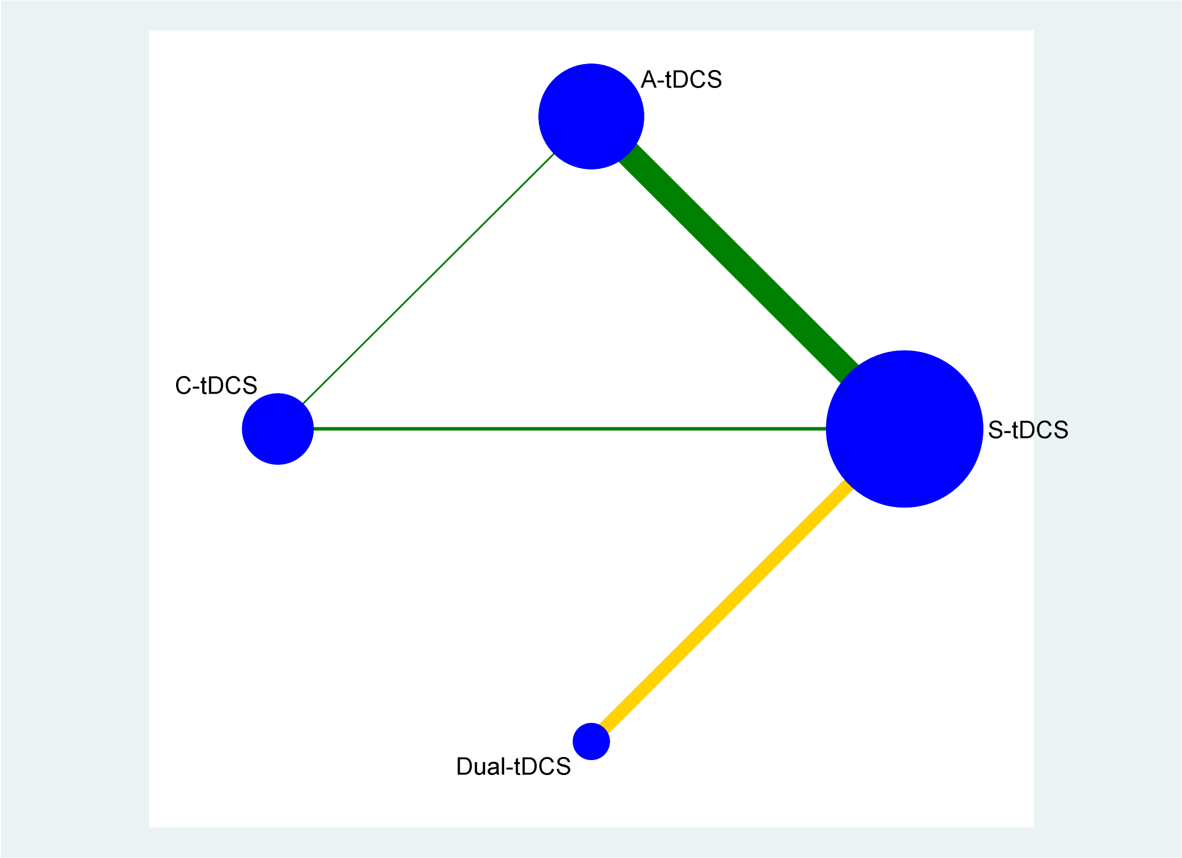

Figure 4: Network graph of the safety of tDCS (measured by number of dropouts and adverse events) after stroke. The thicker the blue bubble, the higher the sample size of the corresponding intervention and the thicker the edge, the lower the standard error of this comparison. Colors of edges indicate risk of bias for this comparison (green = low, yellow = unclear and red = high risk of bias).
