# Supplementary material for: Transcranial direct current stimulation (tDCS) for improving aphasia after stroke: a systematic review with network meta-analysis of randomized controlled trials
Source: J Neuroeng Rehabil. 2020 Jul 8;17:88. doi: 10.1186/s12984-020-00708-z (PMC7346463; doi:10.1186/s12984-020-00708-z)
Supplement: Supplementary file 5 — Additional file 5. Presentation of outcomes of included studies. [file 12984_2020_708_MOESM5_ESM.pdf]

Additional file 5: presentation of risk of bias and outcome data, by studies included in quantitative synthesis (Meta-Analysis)

tDCS for improving functional communication after stroke

Study	Assessment used	Anodal tDCS		Cathodal tDCS		Dual tDCS		Sham tDCS		Risk of Bias		
		Mean (SD)	Participants, *n*	Mean (SD)	Participants, *n*	Mean (SD)	Participants, *n*	Mean (SD)	Participants, *n*	Sequence generation	Allocation concealment	Blinded outcome assessors
Meinzer et al., 2016	CETI	78.5 (19.6)	13					69.1 (20.9)	13	Unclear	Unclear	Low
Spielmann et al., 2016	ANELT	33 (12.5)	26					32 (11.1)	32	Low	Low	Low
Turkeltaub et al., 2017	CETI					6.6 (2)	18	6.4 (1.9)	10	Unclear	Unclear	Low

ANELT: Amsterdam-Nijmegen Everyday Language Test, CETI: Communicative Effectiveness Index, SD: standard deviation, tDCS: transcranial direct current stimulation

tDCS for improving performance in naming nouns after stroke

Study	Assessment used	Anodal tDCS		Cathodal tDCS		Dual tDCS		Sham tDCS		Risk of Bias		
		Mean (SD)	Participants, *n*	Mean (SD)	Participants, *n*	Mean (SD)	Participants, *n*	Mean (SD)	Participants, *n*	Sequence generation	Allocation concealment	Blinded outcome assessors
Fiori et al., 2013	Performance in naming nouns	8.8 (5.7)	4					0 (0)	3	Unclear	Unclear	Low
Flöel et al., 2011	Performance in naming nouns	94.5 (3.7)	4	85.8 (13.2)	4			69.8 (46.7)	4	Low	Unclear	Low
Fridriksson et al., 2018	Performance in naming nouns	13.9 (14)	34	8.2 (13.8)	40					Low	Low	Low
Kang et al., 2011	Performance in naming nouns			3.8 (5.8)	5			1.4 (1.9)	5	Unclear	Unclear	Low
Marangolo et al., 2013b	Performance in naming nouns					7.5 (17.4)	4	20 (15.6)	4	Unclear	Unclear	Low
Meinzer et al., 2016	Performance in naming nouns	27.9 (14.9)	13					16.7 (16.4)	13	Unclear	Unclear	Low
Monti et al., 2008	Performance in naming nouns	0 (0)	2	1 (1.4)	2			0.25 (0.5)	4	Low	Low	Low
Polanowska et al., 2013	Performance in naming nouns	11.8 (6.2)	18					7.3 (4.6)	19	Low	Low	Low

Spielmann et al., 2016	Performance in naming nouns	6.5 (3.8)	26					4.7 (4.37)	32	Low	Low	Low
Turkeltaub et al., 2017	Performance in naming nouns					2.8 (5.5)	23	2.8 (20.5)	14	Unclear	Unclear	Low
You et al., 2011	Performance in naming nouns	7.1 (6.2)	7	11.1 (16.2)	7			5.4 (10.3)	7	Unclear	Unclear	Low

SD: standard deviation, tDCS: transcranial direct current stimulation

tDCS for improving performance in naming verbs after stroke

Study	Assessment used	Anodal tDCS		Cathodal tDCS		Dual tDCS		Sham tDCS		Risk of Bias		
		Mean (SD)	Participants, *n*	Mean (SD)	Participants, *n*	Mean (SD)	Participants, *n*	Mean (SD)	Participants, *n*	Sequence generation	Allocation concealment	Blinded outcome assessors
Fiori et al., 2013	Performance in naming verbs	1.3 (1.3)	4					0 (4.4)	3	Unclear	Unclear	Low
Marangolo et al., 2013b	Performance in naming verbs					13.1 (17.9)	4	15.9 (21)	4	Unclear	Unclear	Low
Marangolo et al., 2018	Performance in naming verbs			25 (17.9)	3			17 (11.8)	3	Low	Low	Low

SD: standard deviation, tDCS: transcranial direct current stimulation

Safety of tDCS for improving functional communication or naming performance after stroke, measured by number of dropouts and adverse events

Study	Anodal tDCS		Cathodal tDCS		Dual tDCS		Sham tDCS		Risk of Bias		
	Events, *n*	Participants, *n*	Events, *n*	Participants, *n*	Events, *n*	Participants, *n*	Events, *n*	Participants, *n*	Sequence generation	Allocation concealment	Blinded outcome assessors
Dos Santos et al., 2017					0	5	0	4	High	Unclear	Unclear
Fiori et al., 2013	0	2					0	3	Unclear	Unclear	Low
Flöel et al., 2011	0	4	0	4			0	4	Low	Unclear	Low
Fridriksson et al., 2018	3	34					6	40	Low	Low	Low
Kang et al., 2011	0	5					0	5	Unclear	Unclear	Low
Marangolo et al., 2011	0	2					0	1	Low	Unclear	Low

Marangolo et al., 2013b					0	8	0	8	Unclear	Unclear	Low
Marangolo et al., 2018			0	3			0	3	Low	Low	Low
Meinzer et al., 2016	0	13					0	13	Unclear	Unclear	Low
Monti et al., 2008	0	2	0	2			0	4	Low	Low	Low
Polanowska et al., 2013	0	14					2	12	Low	Low	Low
Spielmann et al., 2016	1	26					2	32	Low	Low	Low
Turkeltaub et al., 2017					0	24	0	14	Unclear	Unclear	Low
Volpe et al., 2014	0	8					0	7	Unclear	Unclear	Low
You et al., 2011	3	10	4	11			5	11	Unclear	Unclear	Low

tDCS: transcranial direct current stimulation

Studies, which examined tDCS for improving functional communication or language function after stroke and could not be included in quantitative synthesis due to missing information

**Study**	**Assessment used**	**Anodal tDCS**	**Cathodal tDCS**	**Dual tDCS**	**Sham tDCS**	**Risk of Bias**		
		Participants, *n*	Participants, *n*	Participants, *n*	Participants, *n*	Sequence generation	Allocation concealment	Blinded outcome assessors
Baker et al., 2010	Naming performance	10			10	Low	Unclear	Low
Branscheidt et al., 2018	Naming performance, reaction time	16			16	Low	Unclear	Low
Guillouet et al., 2020	Functional communication, naming performance			10	10	Low	Low	Low
Marangolo et al., 2013a	Naming performance	24 (12+12)			12	Low	Low	Low
Marangolo et al., 2013c	Naming performance, reaction time	10 (5+5)			5	Unclear	Unclear	Unclear
Pestalozzi et al., 2018	Naming performance, verbal fluency, word repetition	14			14	Unclear	Low	Low

Rosso et al., 2014	Naming performance, interhemispheric balance		25	25	Unclear	Unclear	Unclear
Shah-Basak et al., 2015	WAB	14 (7+7)	14 (7+7)	14 (7+7)	Unclear	Unclear	Low
Spielmann et al., 2018	Naming performance	13		13	Unclear	Unclear	Low
Vila-Nova et al., 2019	Naming performance, syllable repetition	12		12	Unclear	Unclear	blind

SD: Standard deviation, tDCS: transcranial direct current stimulation, WAB: Western Aphasia Battery
