# Supplementary material for: Transcranial direct current stimulation (tDCS) for improving aphasia after stroke: a systematic review with network meta-analysis of randomized controlled trials
Source: J Neuroeng Rehabil. 2020 Jul 8;17:88. doi: 10.1186/s12984-020-00708-z (PMC7346463; doi:10.1186/s12984-020-00708-z)
Supplement: Supplementary file 6 — Additional file 6. Results of subgroup analyses. [file 12984_2020_708_MOESM6_ESM.pdf]

Additional file 6: results of prespecified subgroup analyses

Presentation of network structure

Figure 1 shows a network graph comparing anodal, cathodal, and dual tDCS and their corresponding stimulation location with their control interventions for improving functional communication after stroke. Figure 2 shows a network graph comparing anodal, cathodal, and dual tDCS and their corresponding stimulation location with their control interventions for improving language function (naming nouns) after stroke and Figure 3 for improving naming verbs. Figure 4 shows a network graph comparing tDCS and its corresponding stimulation location with their control interventions regarding safety (measured by the number of dropouts and adverse events).

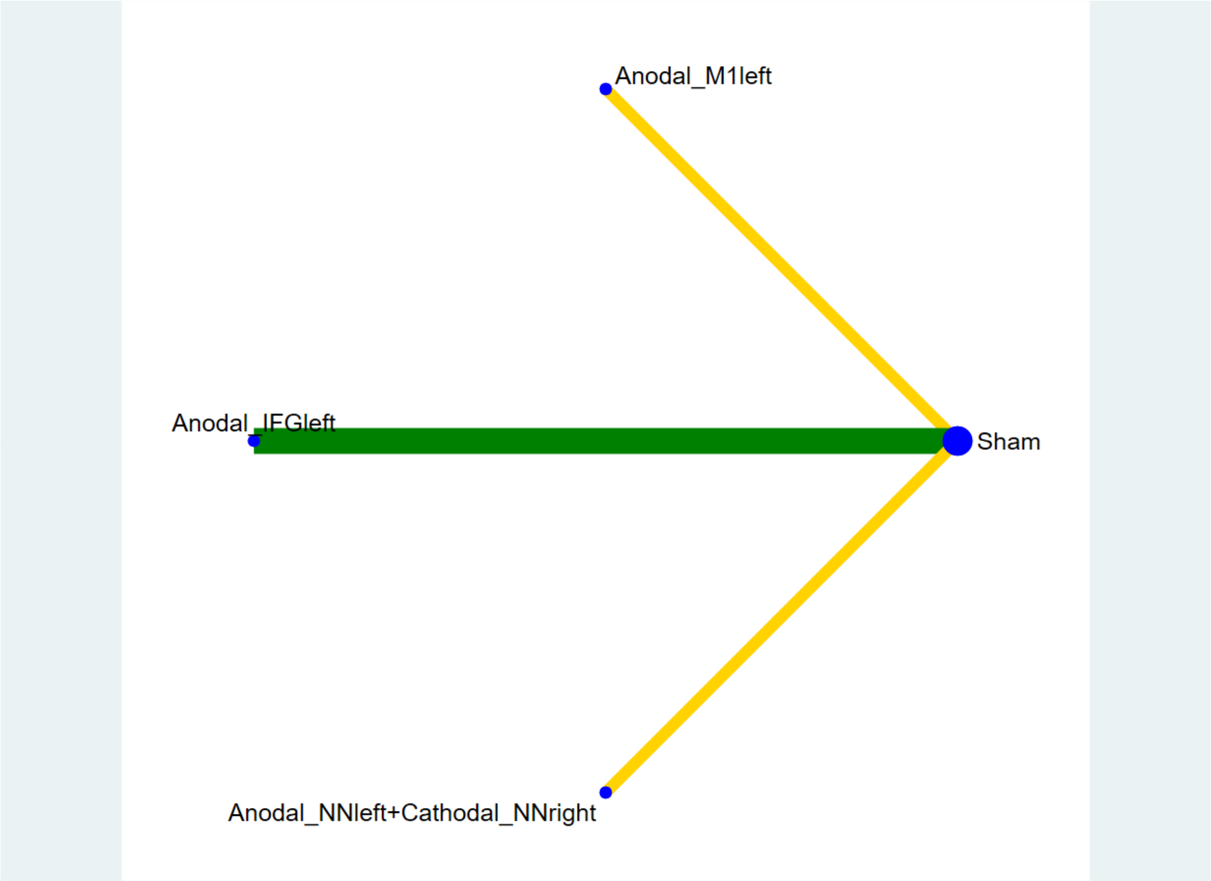

Figure 1: Network graph of tDCS and corresponding stimulation location for improving functional communication in people with aphasia after stroke. The thicker the blue bubble, the higher the sample size of the corresponding intervention and the thicker the edge, the lower the standard error of this comparison. Colors of edges indicate risk of bias for this comparison (green = low, yellow = unclear and red = high risk of bias). IFG: inferior frontal gyrus, M1: primary motor cortex

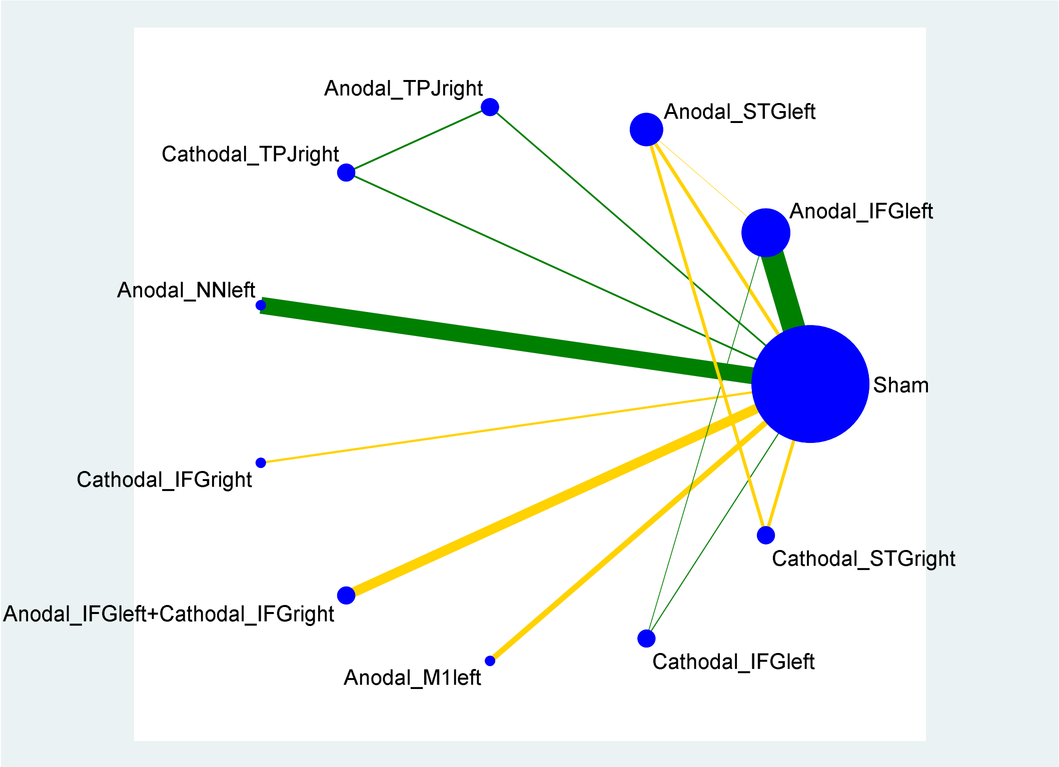

Figure 2: Network graph of tDCS and corresponding stimulation location for improving language function (measured by the performance in naming nouns) in people with aphasia after stroke. The thicker the blue bubble, the higher the sample size of the corresponding intervention and the thicker the edge, the lower the standard error of this comparison. Colors of edges indicate risk of bias for this comparison (green = low, yellow = unclear and red = high risk of bias). IFG: inferior frontal gyrus, M1: primary motor cortex, NN: not named, STG: superior temporal gyrus, TPJ: temporoparietal junction

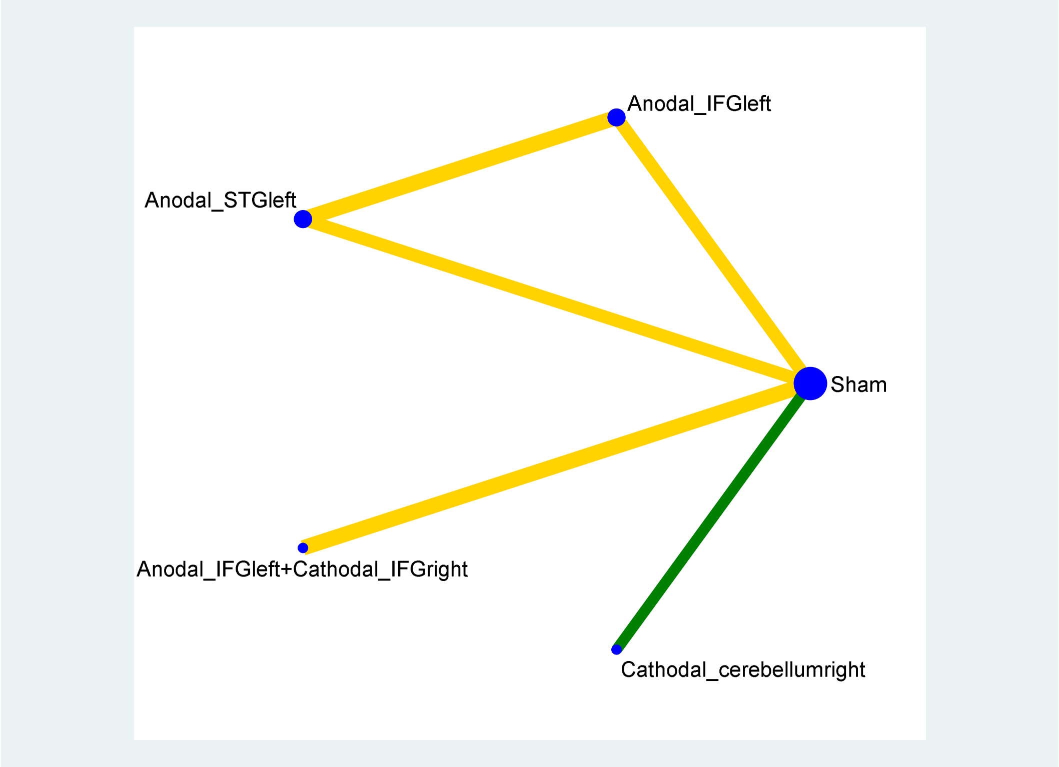

Figure 3: Network graph of tDCS and corresponding stimulation location for improving language function (measured by the performance in naming verbs) in people with aphasia after stroke. The thicker the blue bubble, the higher the sample size of the corresponding intervention and the thicker the edge, the lower the standard error of this comparison. Colors of edges

indicate risk of bias for this comparison (green = low, yellow = unclear and red = high risk of bias). IFG: inferior frontal gyrus, NN: not named, STG: superior temporal gyrus

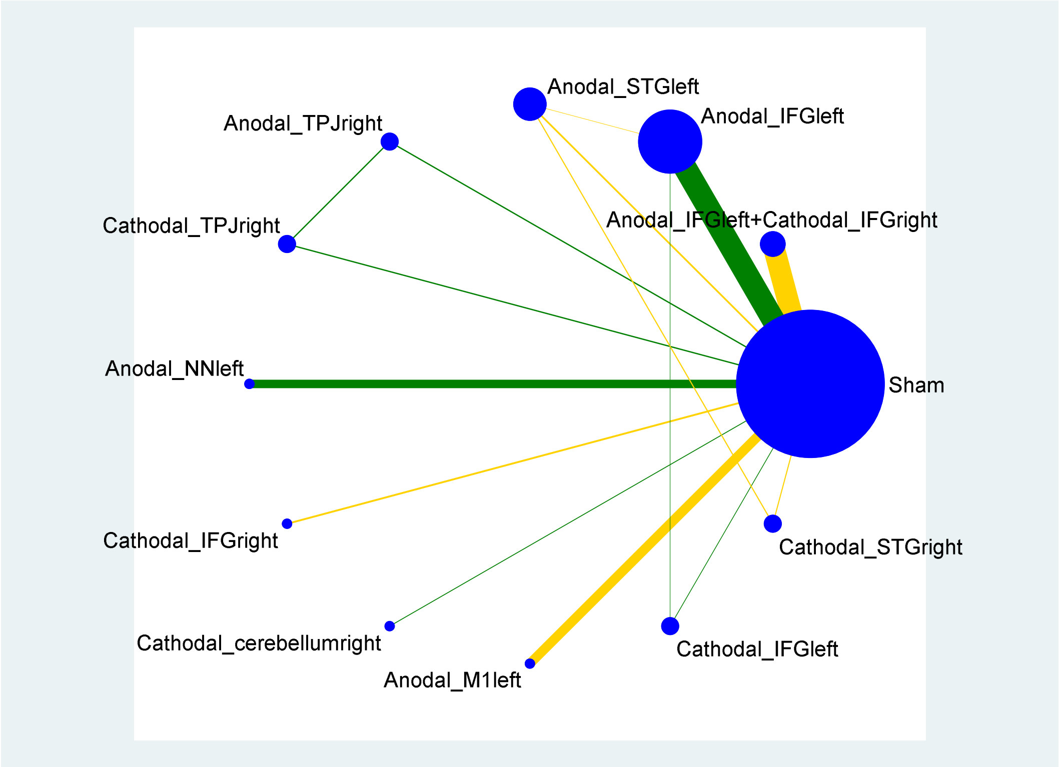

Figure 4: Network graph of the safety of tDCS (measured by number of dropouts and adverse events) and corresponding stimulation location after stroke. The thicker the blue bubble, the higher the sample size of the corresponding intervention and the thicker the edge, the lower the standard error of this comparison. Colors of edges indicate risk of bias for this comparison (green = low, yellow = unclear and red = high risk of bias). IFG: inferior frontal gyrus, M1: primary motor cortex, NN: not named, STG: superior temporal gyrus, TPJ: temporoparietal junction

Synthesis of results

Figure 5 provides a comparison of effect estimates of different tDCS interventions for improving functional communication. Table 1 shows the ranking of the treatments by SUCRA.

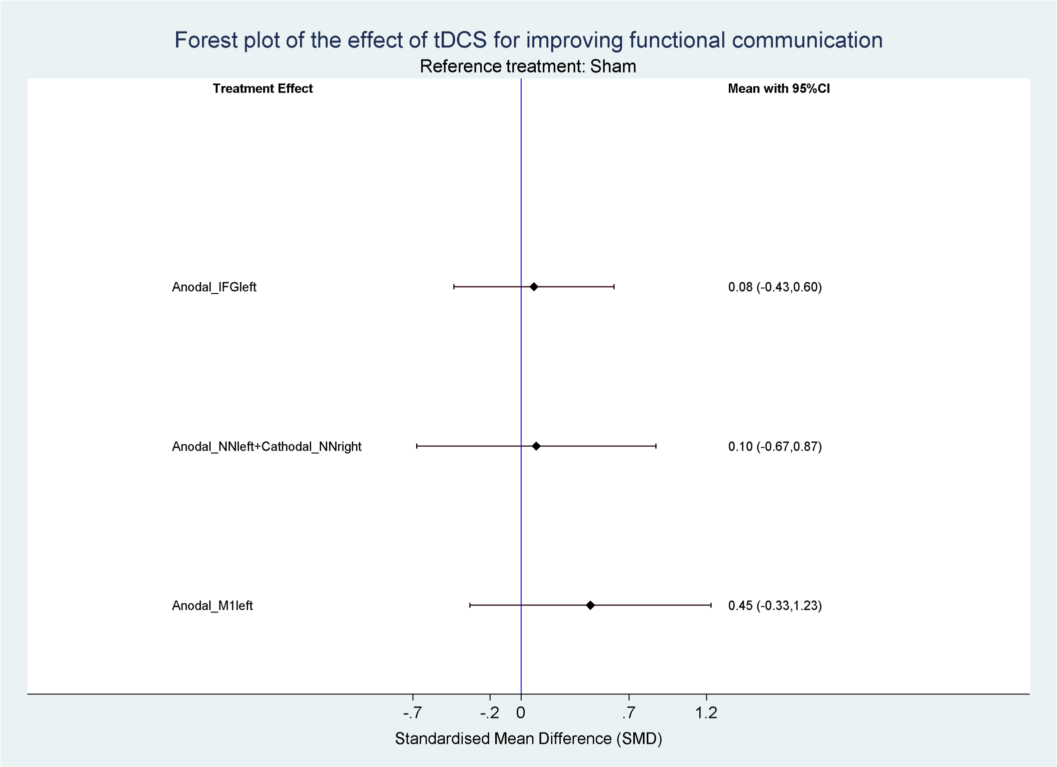

Figure 5: Forest plot of tDCS for improving functional communication in people with aphasia after stroke (3 studies with 112 participants). CI = confidence interval, IFG: inferior frontal gyrus, M1: primary motor cortex. Sham is the reference category.

Table 1: treatment rankings by SUCRA of tDCS for improving functional communication

**Treatment**	**SUCRA**
Anodal M1 left	0.80
Anodal IFG left	0.46
Anodal NN left+cathodal NN right	0.45
Sham	0.29

Treatments are listed in order of relative ranking. The SUCRA, ranging from 0 to 100, describe the percentage efficacy of each individual intervention in comparison with an 'ideal' treatment. IFG: inferior frontal gyrus, M1: primary motor cortex

Effect estimates of different tDCS interventions for improving performance in naming nouns can be found in Figure 6. Table 2 shows the ranking of the treatments by SUCRA.

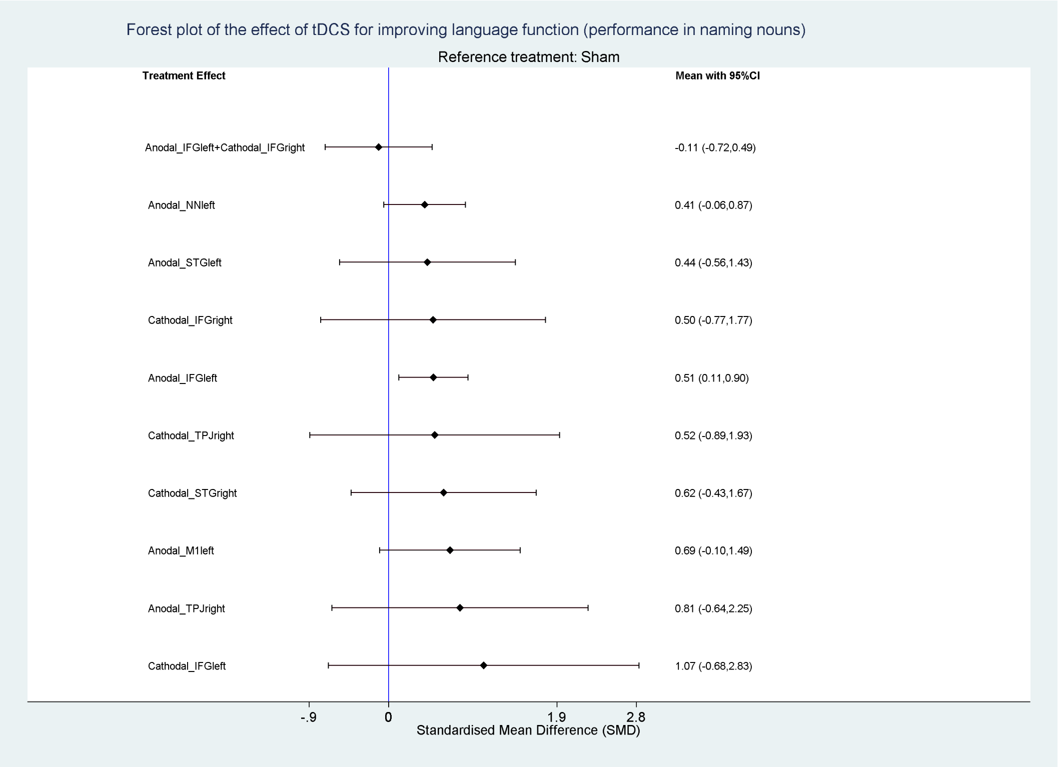

Figure 6: Forest plot of tDCS and corresponding stimulation location for improving language function (performance in naming nouns) in people with aphasia after stroke (11 studies with 298 participants). CI = confidence interval, IFG: inferior frontal gyrus, M1: primary motor cortex, NN: not named, STG: superior temporal gyrus, TPJ: temporoparietal junction. Sham is the reference category.

Table 2: treatment rankings by SUCRA of tDCS for improving performance in naming nouns

**Treatment**	**SUCRA**
Cathodal IFG left	0.72
Anodal M1 left	0.65
Anodal TPJ right	0.65
Cathodal STG right	0.59
Anodal IFG left	0.56
Cathodal IFG right	0.52
Cathodal TPJ right	0.51
Anodal NN left	0.48
Anodal STG left	0.48
Sham	0.18

Anodal IFG left+cathodal IFG right	0.16

Treatments are listed in order of relative ranking. The SUCRA, ranging from 0 to 100, describe the percentage efficacy of each individual intervention in comparison with an 'ideal' treatment. IFG: inferior frontal gyrus, M1: primary motor cortex, NN: not named, STG: superior temporal gyrus, TPJ: temporoparietal junction

Effect estimates of different tDCS interventions for improving performance in naming verbs can be found in Figure 7. Table 3 shows the ranking of the treatments by SUCRA.

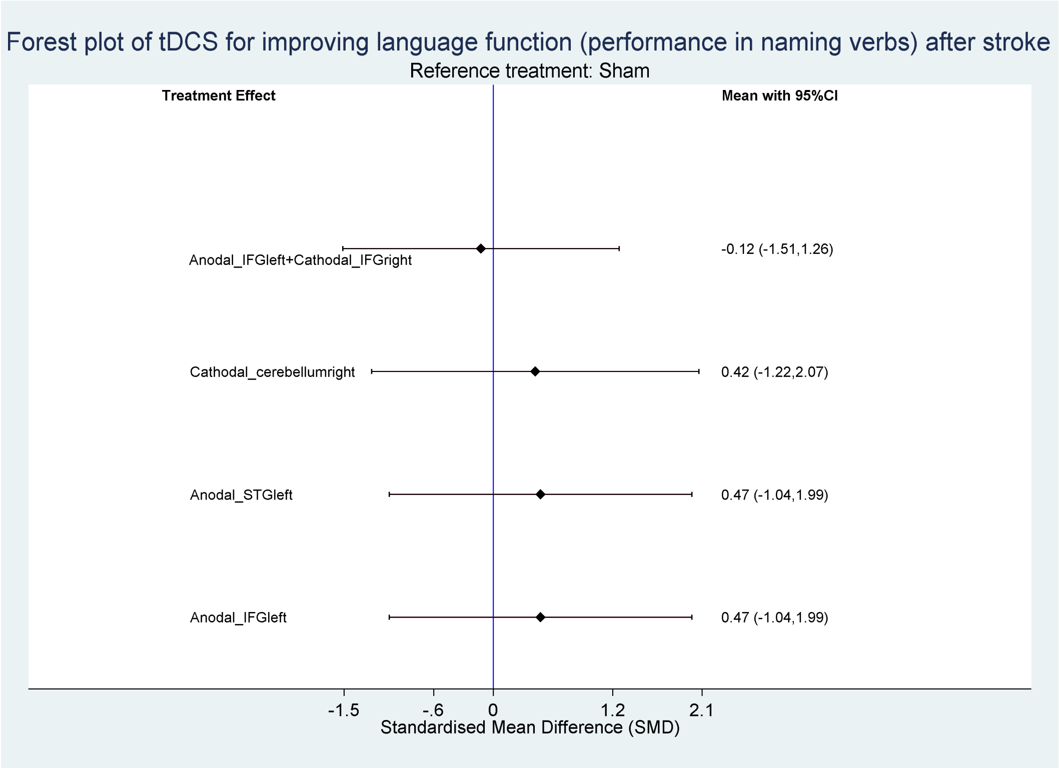

Figure 7: Forest plot of tDCS and corresponding stimulation location for improving language function (performance in naming verbs) in people with aphasia after stroke (3 studies with 21 participants). CI = confidence interval, IFG: inferior frontal gyrus, STG: superior temporal gyrus. Sham is the reference category.

Table 3: treatment rankings by SUCRA of tDCS for improving performance in naming nouns

**Treatment**	**SUCRA**
Anodal STG left	0.63
Anodal IFG left	0.62
Cathodal cerebellum right	0.59
Sham	0.34
Anodal IFG left+cathodal IFG right	0.33

Treatments are listed in order of relative ranking. The SUCRA, ranging from 0 to 100, describe the percentage efficacy of each individual intervention in comparison with an 'ideal' treatment. IFG: inferior frontal gyrus, M1: primary motor cortex, NN: not named, STG: superior temporal gyrus, TPJ: temporoparietal junction

Figure 8 provides a comparison of effect estimates of different tDCS interventions regarding safety. Table 4 shows the ranking of the treatments by SUCRA.

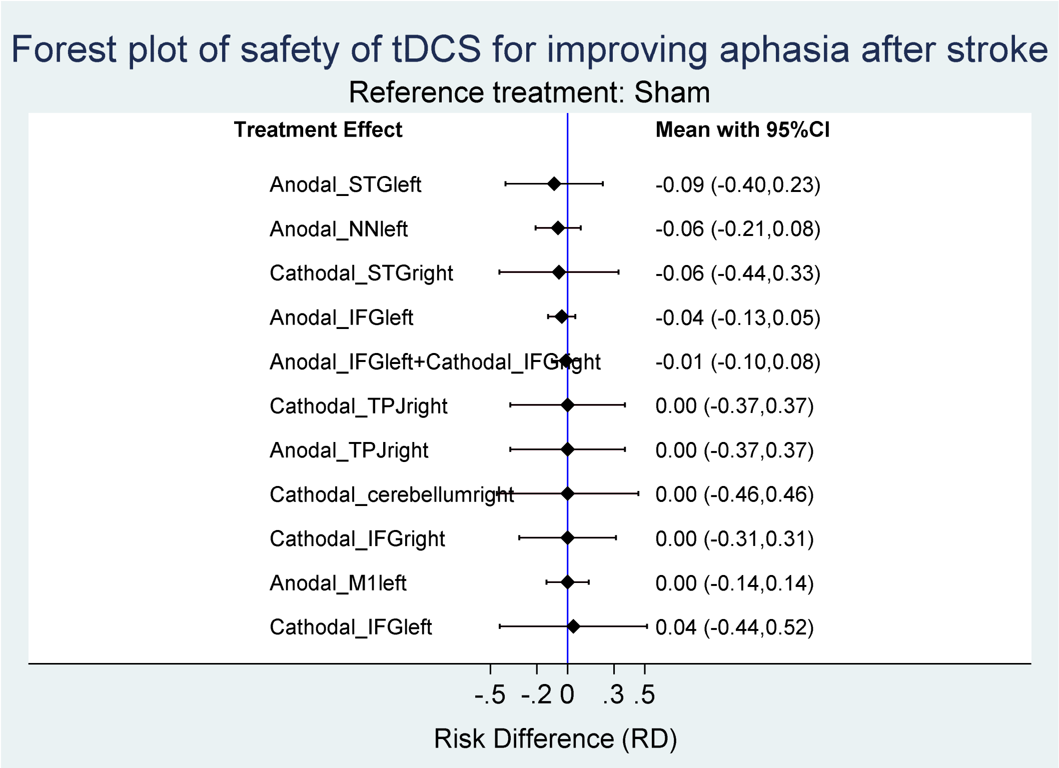

Figure 8: Forest plot of the safety of tDCS for improving functional communication or language function after stroke (15 studies with 338 participants). CI = confidence interval, IFG: inferior frontal gyrus, NN: not named, M1: primary motor cortex, STG: superior temporal gyrus, TPJ: temporoparietal junction. Sham is the reference category; measure of effect is risk difference (RD).

Table 4: treatment rankings by SUCRA of safety of tDCS for improving functional communication or language function

**Treatment**	**SUCRA**
Cathodal IFG left	0.61
Sham	0.58
Anodal M1 left	0.57
Anodal TPJ right	0.55

Cathodal IFG right	0.53
Cathodal TPJ right	0.53
Cathodal cerebellum right	0.52
Anodal IFG left+cathodal IFG right	0.52
Cathodal STG right	0.44
Anodal IFG left	0.43
Anodal NN left	0.38
Anodal STG left	0.35

Treatments are listed in order of relative ranking. The SUCRA, ranging from 0 to 100, describe the percentage efficacy of each individual intervention in comparison with an 'ideal' treatment. IFG: inferior frontal gyrus, M1: primary motor cortex, NN: not named, STG: superior temporal gyrus, TPJ: temporoparietal junction

Exploration for inconsistency

Significant inconsistency, which means disagreement between direct and indirect comparisons, was not observed. For the outcomes functional communication and performance in naming verbs there was no source of inconsistency. Formal testing did not detect statistically significant design inconsistency for the outcomes performance in naming nouns and safety (χ^2=4.46; df=3; p=0.22 and χ^2=0.53; df=3; p=0.91, respectively).
